# Natural products can modulate inflammation in intervertebral disc degeneration

**DOI:** 10.3389/fphar.2023.1150835

**Published:** 2023-02-16

**Authors:** Zongtai Liu, Jiabo Zhu, Haiyan Liu, Changfeng Fu

**Affiliations:** ^1^ Department of Spine Surgery, First Hospital of Jilin University, Changchun, China; ^2^ Department of Orthopedics, Affiliated Hospital of Beihua University, Jilin, China; ^3^ Department of Orthopedics, Baicheng Central Hospital, Baicheng, China

**Keywords:** intervertebral disc, intervertebral disc degeneration, lower back pain, inflammation, natural product

## Abstract

Intervertebral discs (IVDs) play a crucial role in maintaining normal vertebral anatomy as well as mobile function. Intervertebral disc degeneration (IDD) is a common clinical symptom and is an important cause of low back pain (LBP). IDD is initially considered to be associated with aging and abnormal mechanical loads. However, over recent years, researchers have discovered that IDD is caused by a variety of mechanisms, including persistent inflammation, functional cell loss, accelerated extracellular matrix decomposition, the imbalance of functional components, and genetic metabolic disorders. Of these, inflammation is thought to interact with other mechanisms and is closely associated with the production of pain. Considering the key role of inflammation in IDD, the modulation of inflammation provides us with new options for mitigating the progression of degeneration and may even cause reversal. Many natural substances possess anti-inflammatory functions. Due to the wide availability of such substances, it is important that we screen and identify natural agents that are capable of regulating IVD inflammation. In fact, many studies have demonstrated the potential clinical application of natural substances for the regulation of inflammation in IDD; some of these have been proven to have excellent biosafety. In this review, we summarize the mechanisms and interactions that are responsible for inflammation in IDD and review the application of natural products for the modulation of degenerative disc inflammation.

## 1 Introduction

Low back pain (LBP) is a common clinical disease that creates a serious burden on a patient’s life and the social economy. According to previous epidemiological surveys, LBP is a major cause of global disability (Foster et al., 2018). Approximately 80% of people will experience LBP during their lifetime. Worryingly, this proportion is likely to increase as the average life expectancy of the population rises and population aging accelerates (Conway, 2017; Kaye et al., 2022). In the 2016 U.S. Healthcare Spending Survey, low back and neck pain were responsible for the highest healthcare expenditure among 154 diseases, at $134.5 billion (Dieleman et al., 2020). Intervertebral disc degeneration (IDD) is one of the most significant pathogenic factors of LBP, and approximately 40% of LBP cases are caused by IDD (Peng, 2013). IDD is also the pathological basis of many spinal degenerative diseases. Severe IDD can lead to herniation and spinal canal stenosis (Kushchayev et al., 2018; Benzakour et al., 2019). In addition to pain, these diseases may also cause severe sensory and motor dysfunction, further reducing the quality of life of patients and increasing the economic burden (Quaile, 2019).

At present, the clinical treatment of IDD can be divided into non-surgical conservative treatment and surgical treatment (Wu et al., 2020). Conservative treatment is the first choice for most patients. Conservative treatment can be further divided into pharmacological and non-pharmacological treatments. The common medications used for IDD include opioids, anti-inflammatory drugs, muscle relaxants and anticonvulsant/antidepressant drugs (Mohd Isa et al., 2022). The main effect of these drugs is to relieve pain and the further complications caused by pain. Although the short-term analgesic effect of drugs is clear, long-term use will undoubtedly increase the risk of drug addiction and drug abuse and cause damage to the internal organs of patients (Fishbain et al., 1992). The most common non-pharmacological treatments include exercise therapy, massage, acupuncture, and psychological intervention. However, the efficacy of these treatments lacks robust evidence and well-designed cohort studies (Rickers et al., 2021). In addition, there is also a lack of valid comparative studies between different treatment modalities. Surgery is the last choice for patients with severe symptoms and those who fail to respond to conservative treatment. Although the development of new surgical techniques, such as endoscopic surgery, has greatly reduced surgical damage and accelerated postoperative recovery, the damage caused to local tissue and mechanical structures is irreversible, and the corresponding complications are difficult to avoid by simply improving the surgical technique involved (Pan et al., 2020). Furthermore, most existing treatments focus on the relief of existing symptoms, when degeneration is often difficult to reverse. Therefore, effective treatments that can intervene in the early stages of degeneration are urgently needed.

Normal anatomical structure is the basis for maintaining the physiological function of the intervertebral disc (IVD). IVDs are fibrocartilage structures located between adjacent vertebral bodies, and are composed of the nucleus pulposus (NP), annulus fibrosus (AF) and cartilage endplate (CEP) (Mohd Isa et al., 2022). The NP is located at the core of the IVD and is highly hydrated. An abundance of proteoglycans helps to maintain the water content of the IVD which provides sufficient hydrostatic pressure to resist mechanical load (Guerrero et al., 2021). In addition, the NP is also rich in type II collagen; a reduction in type II collagen, along with an increase in type I collagen, is considered to be one of the hallmark changes of disc degeneration (Wu et al., 2016). The AF is mainly composed of multilayer collagen fibers arranged in a regular manner, with a gradual reduction in the content of type I collagen and a gradual increase in the content of type II collagen from the outer layer to the inner layer (Eyre and Muir, 1976; Sloan et al., 2018). The main function of the fibrous ring is to resist local mechanical forces to limit the protrusion of the NP. Degenerative AF mainly manifests as increased small fissures; the accumulation of fissures will eventually lead to the formation of hernias and cause local mechanical load disorder (Torre et al., 2019). The CEP is a layer of hyaline cartilage that separates the bony vertebral body from the IVD and is responsible for distributing pressure (Moon et al., 2013). In addition, due to the avascular structure of the IVD, the CEP is also responsible for providing oxygen and nutrients to the IVD (Urban et al., 2004). CEP degeneration is characterized by calcification, thinning and uneven thickness, thus resulting in an uneven load distribution and limitations in nutrient and oxygen transport (Ashinsky et al., 2020). Due to the fragile nutrient supply and low cell density, the self-repair ability of IVD is extremely limited (Gantenbein et al., 2020).

Existing literature suggests that the pathological process of IDD involves multiple mechanisms (Vergroesen et al., 2015; Mohd Isa et al., 2022). While many mechanisms are being investigated, inflammation has received extensive attention. As a widespread defense mechanism, the inflammatory response caused by different pathological processes has certain commonalities, thus creating more entry points for researchers (Risbud and Shapiro, 2014; Wang et al., 2022c; Li F et al., 2022). Inflammation is intrinsically closely related to many other mechanisms that are known to be associated with IDD, such as cell loss, the reduction of extracellular matrix (ECM), and the dysregulation of functional components (Risbud and Shapiro, 2014; Khan et al., 2017). In addition, inflammation is also closely associated with the production of pain (Lyu et al., 2021). The abundance of natural anti-inflammatory substances provides ample options to modulate inflammation in IDD. Here, we summarize the critical role of inflammation in IDD and their relationships with other mechanisms. Subsequently, we review the application of natural products in the control of inflammation in IDD.

## 2 Relationships between inflammation and IDD

### 2.1 Inflammatory factors and related pathways in IDD

An abundance of previous studies has reported the presence of elevated inflammatory factors in IDD patients, such as interleukins (IL)-1, −6, −8, −12, −17, tumor necrosis factor (TNF)-α, nitric oxide (NO), interferon (IFN)-γ, and prostaglandin E2 (PGE2) (Lyu et al., 2021). Although the detailed inflammatory mechanisms and signaling pathways are still not fully understood, IL-1β and TNF-α may be the upstream factors that drive the inflammatory cascade (Johnson et al., 2015). (Maitre et al., 2005) reported that IL-1β levels increase with the severity of degeneration. In their subsequent study, these authors reported higher levels of IL-1β secretion compared to TNF-α in IDD patients (Le Maitre et al., 2007a). The activation of IL-1β precursor proIL-1β requires inflammasomes and caspase-1; furthermore, the activation of Nod-like receptor protein (NLRP)-3 in inflammasomes has received significant attention. Chen et al. (Chen et al., 2015) further reported that NLRP-3, caspase-1, and IL-1β were positively correlated with IVD tissue degeneration scores. Bioactive TNF-α can be divided into transmembrance TNF-α (mTNF-α) and secreted TNF-α (sTNF-α) (Jang et al., 2021). The mTNF-α is cleaved by TNF-α-converting enzyme to form sTNF-α. Similarly, TNF-α receptors can be divided into tumor necrosis factor receptor (TNFR) 1 and TNFR2. Both of these receptors can bind to mTNF-α, whereas sTNF-α can only bind to TNFR 1 (Pobezinskaya and Liu, 2012; Kalliolias and Ivashkiv, 2016).

The nuclear factor kappa-B (NF-κB) and mitogen-activated protein kinase (MAPK) signaling pathway play essential roles in IDD inflammation (Zhang et al., 2021a). The NF-κB signaling pathway is widely present in animals and is an important mechanism by which cells respond to external stimuli (Capece et al., 2022). A number of inflammatory factors, such as IL-1β, −6, −8, −12, and TNF-α, can activate this pathway (DiDonato et al., 2012; Hoesel and Schmid, 2013). Normally, NF-κB is bound to the inhibitor of kappa B (IκB). When stimulated, IκB kinase is activated, IκB is degraded, and the expression of target genes is regulated by the nuclear entry of NF-κB. At the same time, free NF-κB stimulates the secretion of new IκB and re-inhibits the activity of NF-κB (Williams and Gilmore, 2020). The MAPK signaling pathway is another pathway that plays a major role in the response of eukaryotic cells to external stimuli (Atay and Skotheim, 2017). The MAPK signaling pathway is highly conserved and has been demonstrated in four different subfamilies in mammals, including extracellular signal-regulated kinases (ERKs), ERK5, c-Jun NH2-terminal kinases (JNKs), and p38 isoforms (p38s) (Cargnello and Roux, 2011). The MAPK signaling pathway follows a pattern of tertiary kinases, including MAPK kinase kinase, MAPK kinase, and MAPK, which are activated in sequence (Burotto et al., 2014). The toll-like receptor (TLR) signaling pathway is an important pathway involved in immune regulation, and has been recently found to play a role in IDD inflammation (Klawitter et al., 2014). TLR2 and TLR4 have received the most extensive study in IDD, and their activation can upregulate the expression of a variety of inflammatory factors (Bisson et al., 2021). Quero et al. (Quero et al., 2013) reported the activation of TLR2 by a hyaluronic acid fragment produced in IDD. In another study, Rajan et al. (Rajan et al., 2013) successfully induced an inflammatory response that eventually led to IDD by activating TLR4. In recent years, infection with *Propionibacterium acnes* has been identified as one of the factors contributing to IDD and may be associated with more pronounced LBP symptoms. Jiao et al. (Jiao et al., 2019) reported that *Propionibacterium acnes* upregulated IL-8 secretion in NP cells by stimulating the TLR-2/NF-κB p56 pathway. In their subsequent study, these authors revealed the association of TLR2 and NF-κB p65/JNK pathways with nerve growth factor (NGF), a key pro-algesic factor (Jiao et al., 2022).

Gaining a deeper understanding of inflammatory mechanisms will yield a large number of potential targets for controlling inflammation in IDD, but screening for the most effective and safe targets still requires extensive follow-up experiments. In addition, although the technology to produce animal models of IDD is well established, the inherent differences between animals and humans still need to be considered (Lyu et al., 2021; Zhu et al., 2022).

### 2.2 Synergistic effects of inflammation and other mechanisms

IDD involves multiple mechanisms that interact to form a vicious cycle; inflammation plays a role in many of these mechanisms. Modulating inflammation in IDD is expected to regulate other mechanisms, thus slowing or even reversing degeneration.

A sufficient number of functional cells is essential to maintain the normal function and metabolism of IVDs. Higher rates of cell senescence, apoptosis and pyroptosis have been reported in IDD, thus leading to a reduction in the quantity of functional cells in IVD (Zhang et al., 2020; Zhang et al., 2021b; Luo et al., 2022). In addition, lower cell density leads to a greater sensitivity to cell depletion. Inflammation plays a crucial role in promoting cell senescence, apoptosis and pyroptosis. Li et al. (Li et al., 2019) cultured NP cells in a medium containing IL-1β and TNF-α; the inflammation group showed higher expression levels of cell senescence markers (β-galactosidase, p16 and p53) and reduced the activity of telomerase compared with a control group without inflammatory factors. Reactive oxygen species (ROS), another important product of inflammation, is also thought to be closely related to accelerated cell senescence (Kim et al., 2009). In addition, ROS are also involved in the activation of the NF-κB and MAPK signaling pathways which can lead to pro-inflammatory effects (Cao et al., 2022). Apoptosis and pyroptosis are different forms of programmed cell death. Jiang et al. (Jiang et al., 2019) reported an increase in apoptosis rate, caspase-3 activity, and the mRNA expression of apoptosis-related molecules, such as caspase-3 and cleaved caspase-3, in NP cells in response to IL-1β stimulation. IL-1β stimulation can also cause mitochondrial oxidative damage and activate NLRP-3, eventually leading to pyroptosis (Ma et al., 2022). Interestingly, Tang et al. (Tang et al., 2021) reported that infection with *Propionibacterium acnes* similarly resulted in the overexpression of ROS and NLRP3, thus resulting in a high rate of pyroptosis in NP cells. Similarly, several previous studies have demonstrated the pro-active effects of TNF-α on apoptosis and pyroptosis (Yu et al., 2018; Qiu et al., 2019; Zhai et al., 2022).

The ECM represents the external environment that is responsible for the survival of IVD cells, and plays a key role in the exchange of cellular information. Indeed, the dysregulation of ECM anabolism and catabolism, and the absence of crucial components, such as proteoglycans and type II collagen, are typical pathological changes in IDD. Matrix metalloproteinases (MMPs), and a disintegrin and metalloproteinase with thrombospondin motifs (ADAMTSs), are two families of enzymes that regulate the ECM (Kibble et al., 2022). Inflammatory factors not only accelerate the catabolism of key substances, but also downregulate their source synthesis and expression. Several previous studies have demonstrated that IL-1β reduces the expression of type II collagen and proteoglycan and elevates the expression of MMP-1, -3, -9, -10, −13, ADAMTS-4, and -5 (Le Maitre et al., 2007b; Kim et al., 2013a; Fang et al., 2018). The expression of these enzymes accelerates ECM breakdown. In addition to IL-1β, Séguin et al. (Séguin et al., 2005) also reported that TNF-α increased the expression levels of MMP-1, -3, -13, ADAMTS-4, and -5, and reduced the expression of type II collagen and proteoglycan genes in NP cells. The development of proteomics and transcriptomics is expected to reveal more detailed changes in the expression and synthesis of substances at the spatiotemporal level (Eckersley et al., 2021; Kibble et al., 2022). Overall, the close relationship between inflammation and other mechanisms further indicates that controlling inflammation has great potential for the treatment of IDD.

## 3 The application of natural products to control inflammation

Seeking suitable anti-inflammatory substances is an important step in controlling inflammation in IDD. Natural products (animals, plants and microorganisms) are significant sources of anti-inflammatory agents and provide ample options for the selection of anti-inflammatory substances. Many natural anti-inflammatory substances have achieved promising results from *in vitro* or *vivo* experiments with good levels of safety. At present, the anti-inflammatory substances used in IDD are mainly derived from animals and plants. Jenab et al. (2020) provide a detailed review of the anti-inflammatory substances produced by microorganisms, although these agents have yet to be applied for the treatment of IDD.

### 3.1 Natural anti-inflammatory products derived from animals

**FIGURE 1:**
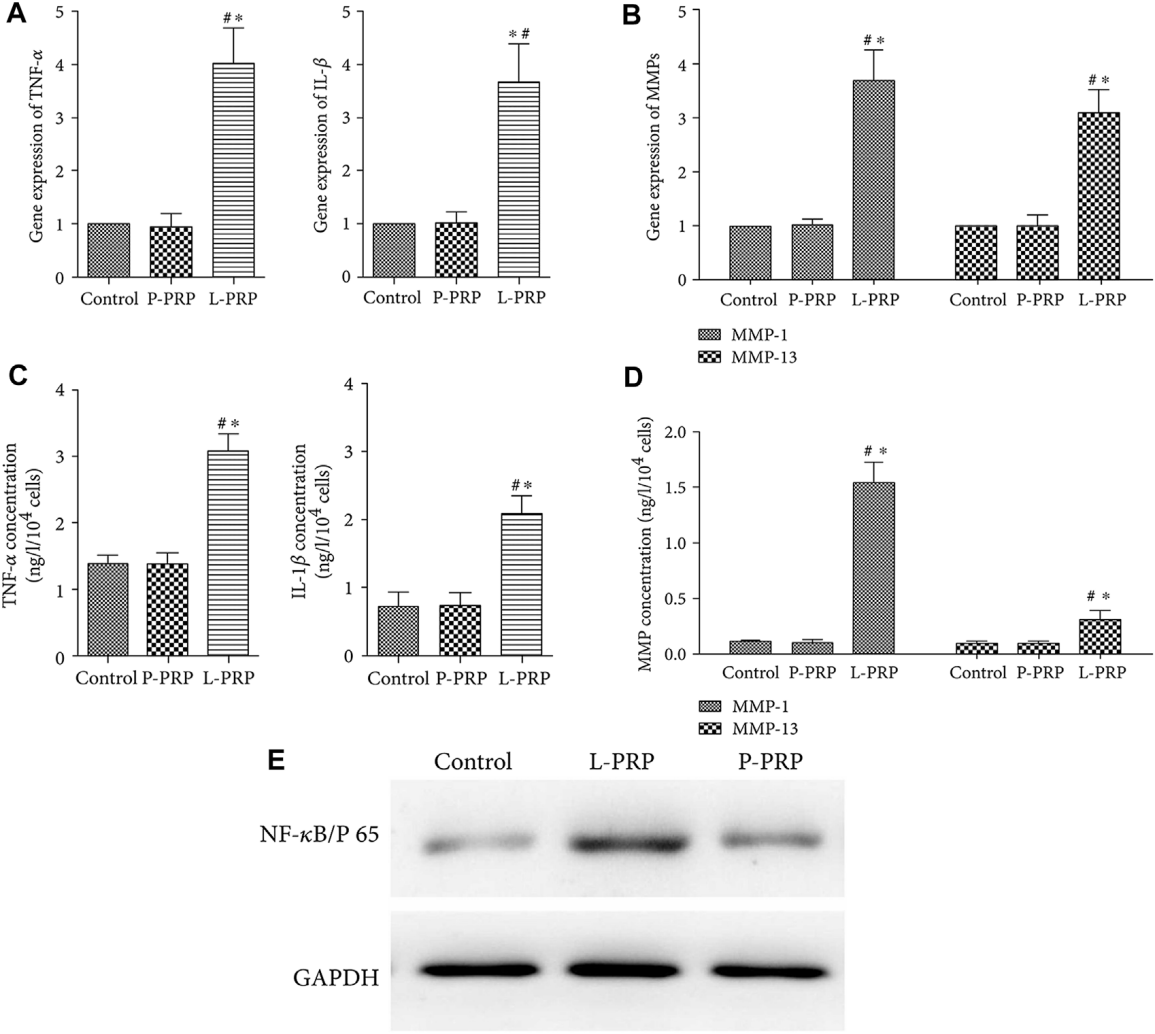
A comparison of the gene expression of inflammatory factors between groups with different leukocyte levels **(A)**. A comparison of gene expression of MMPs between groups with different leukocyte levels **(B)**. A comparison of the concentrations of inflammatory factors between groups with different leukocyte levels **(C)**. A comparison of the concentrations of MMPs between groups with different leukocyte levels **(D)**. West-blot comparison of the production of NF-κB/p65 between groups with different leukocyte levels **(E)**. “*” *p* < 0.05 compared to P-PRP or L-PRP with controls, “#” *p* < 0.05 compared to L-PRP with P-PRP. Reproduced with permission from a previous publication (Jia et al., 2018).

Previous research has shown that a negative feedback mechanism is involved in the process of inflammation in a range of animals (Yoshimura et al., 2003; Afonina et al., 2017). Extracting anti-inflammatory substances secreted by animals is an important method used to acquire anti-inflammatory substances. Platelet-rich plasma (PRP) is a type of autologous blood extract that has been used extensively in clinical practice because of its good tissue repair ability and very low immunogenicity (Chang et al., 2020). In recent years, the anti-inflammatory effects of PRP have gradually received attention. There are key differences in the equipment used, and the processes used to prepare PRP; furthermore, the presence of a large number of leukocytes in PRP may further aggravate the degree of inflammation. Wang et al. (2018b) reported the differential therapeutic effects caused by NP stem cells with leukocyte-platelet-rich PRP (L-PRP) and pure PRP (P-PRP) without leukocytes. These results indicated that the expression levels of inflammatory factors and genes promoting ECM catabolism were upregulated in the L-PRP group, while the expression levels of genes related to ECM anabolism and type II collagen were upregulated in the P-PRP group. Jia et al. (Jia et al., 2018) further found that L-PRP enhanced the activation of NF-κB pathway and upregulated the expression of TNF-α and IL-1 β (Figure 1). More recently, Qian et al. (2022) demonstrated that exosomes derived from PRP could regulate inflammation in IDD by regulating ubiquitination and the autophagic degradation of NLRP-3.

**FIGURE 2:**
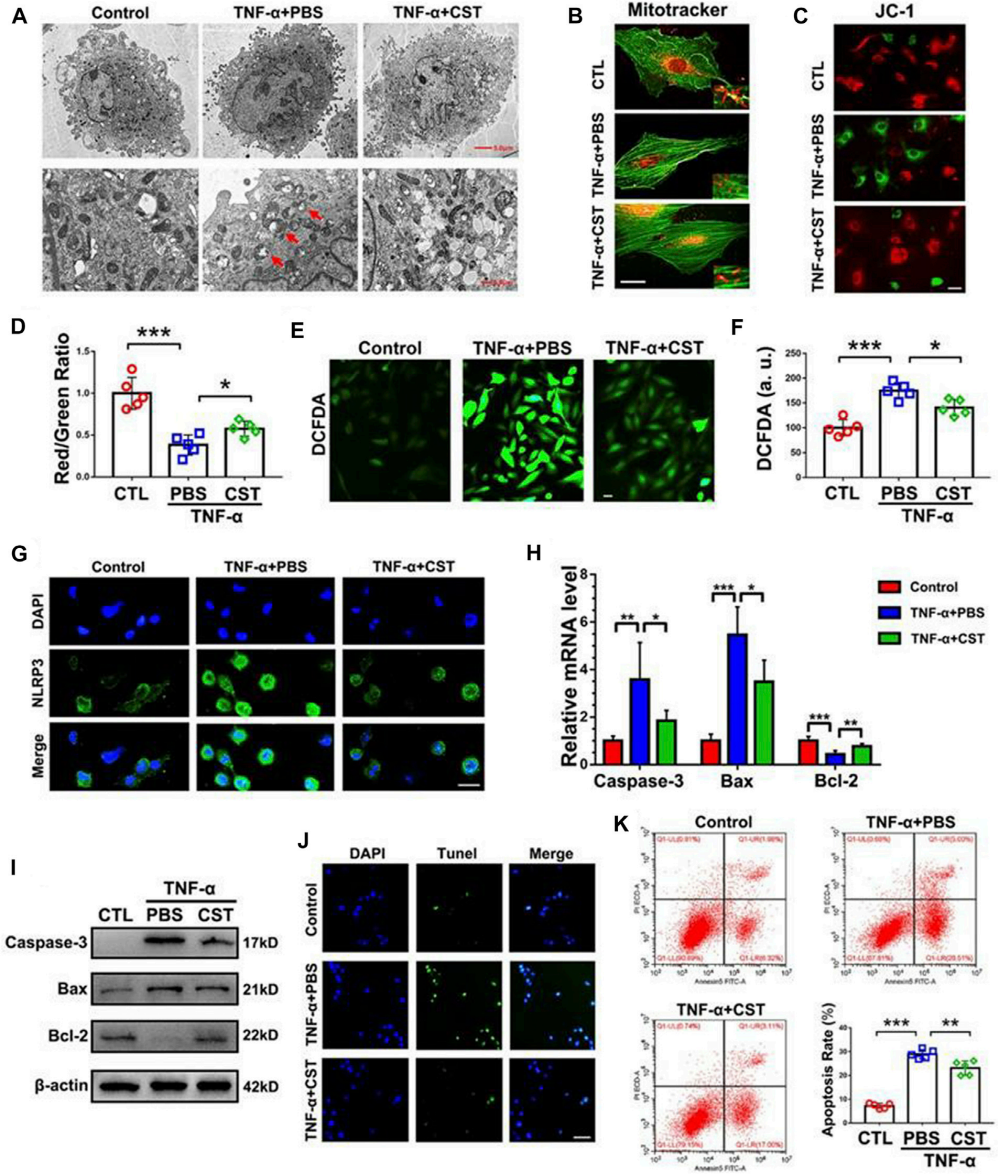
Comparison of human NP cells between groups under transmission electron microscopy **(A)** MitoTracker staining for mitochondria and phalloidin for cytoskeleton **(B)**, scale bar = 10 μm. Comparison of JC-1 assay images between different groups **(C)**, scale bar = 20 μm. Quantitative comparison of red and green fluorescence in JC-1 assays **(D)**. Images of iNOS as determined by DCFDA staining **(E)**, scale bar = 20 μm. Quantitative analysis of iNOS content **(F)**. Comparison of NLRP3 staining in different groups **(G)**, scale bar = 20 μm. Comparison of the caspase-3, Bax, and Bcl-2 mRNA levels in different groups **(H)**. Western blot analysis of caspase-3, Bax and Bcl-2 in different groups **(I)**. Images of TUNEL staining in different groups **(J)**, scale bar = 100 μm. Comparison of the results of the number of apoptotic NPs, as measured by flow cytometry **(K)**. “*” *p* < 0.05, “**” *p* < 0.01 and “***” *p* < 0.001. CST corticostatin. Reproduced with permission from a previous publication (Zhao et al., 2020).

Some cytokines can also be used to regulate inflammation in IDD. For example, Li et al. (2014) treated degenerated NP cells with IL-10 and transforming growth factor (TGF)-β and observed a significant downregulation of IL-1β and TNF-α expression. In another study, Zhang et al. (2017) found that TGF-β1 alleviated inflammation and pain in a rat model of IDD by downregulating the expression of chemokine CCL3/4 *via* the ERK signaling pathway. Growth differentiation factors (GDF) belong to the TGF superfamily. The treatment of rabbit NPs with GDF-6 significantly reduced the secretion of IL-6 and TNF-α and alleviated pain symptoms (Miyazaki et al., 2018). Similarly, Shen et al. (Shen et al., 2018) reported that GDF-5 reduced the expression levels of multiple inflammatory factors and prevented activation of the NF-κB signaling pathway. IL-4 is considered as a cytokine that can exert anti-inflammatory functions. Hou et al. (Kedong et al., 2020) demonstrated that IL-4 reduced the gene expression levels of IL-6, -8, -12, and IFN-β, and ultimately reduced the release of IL-6 and IL-8 proteins. An imbalance between IL-1 and the IL-1 receptor antagonist (IL-1ra) is considered to be one of the factors that promotes inflammation. The sustained release of IL-1ra hydrogel microspheres has been reported to reduce the mRNA levels of IL-1β, IL-6, inducible nitric oxide synthase (iNOS) and other inflammatory mediators (Gorth et al., 2012). Coenzyme Q10 (Co-Q10) is an important substance involved in the electron transport chain and is now available as a common dietary supplement (Arenas-Jal et al., 2020). Wang et al. (2018c) described the inhibitory effects of Co-Q10 on IL-1β-induced multiple downstream inflammatory factors such as IL-6, TNF-α and iNOS. LIM mineralization protein (LMP)-1 is an intracellular protein that regulates bone and cartilage production. Liu et al. (2010) reported that LMP-1 inhibited the NF-κB pathway and significantly downregulated NO production and iNOS expression. In a subsequent study, LMP-1 was further found to reduce apoptosis in IDD by inhibiting the NF-κB pathway (Liu et al., 2020). A previous study showed that the removal of the pineal gland from chickens exacerbated the progression of IDD (Turgut et al., 2006). Qiu et al. (2022) reported that melatonin secreted from the pineal gland could inhibit activation of the TNF-α-induced NF-κB pathway and thus alleviate the progression of degeneration. In addition, these authors also observed the reduced expression of melatonin membrane receptors in degenerative NP tissues. In a previous study, (Li et al., 2018) reported the negative feedback regulation of Wnt5 on TNF-α-induced inflammation. Similarly, lactoferrin has been shown to possess anti-inflammatory, antibacterial, and antitumor properties. Kim et al. (2013b) reported the inhibitory effect of bovine lactoferrin on IL-6, TLR-2, -4, and iNOS. As a cyclic peptide, corticosteroid plays an integral role in many physiological and pathological processes. Zhao et al. (2020) found that the NP cells of IDD patients exhibited reduced levels of corticostatin expression, and that corticostatin-knockout mice showed faster disc degeneration and greater apoptosis. Exogenous corticostatin has been shown to effectively inhibit initiation of the NLRP3 and NF-κB pathway to resist degeneration (Figure 2).

**FIGURE 3:**
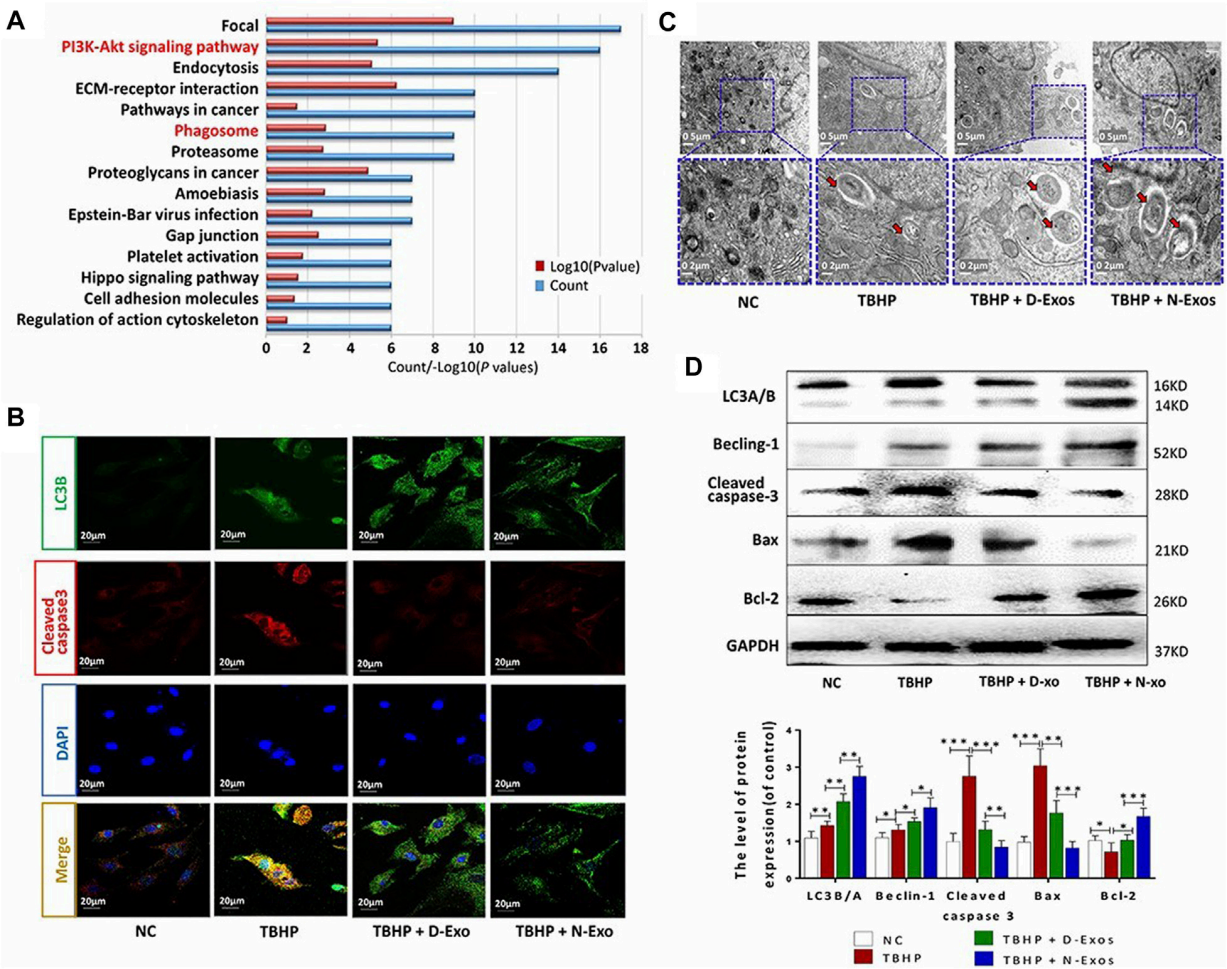
Analysis of the enrichment of differential proteins in exosomes derived from normal CEP stem cells (N-exons) and exosomes derived from degenerate CEP stem cells (D-exons) using KEGG **(A)** Immunofluorescence images of LC3-B and cleaved caspase-3 in NP cells **(B)** Autophagosomes in each group were observed by transmission electron microscopy **(C)** Western blots and quantitative analysis of LC3B/A, Beclin-1, cleaved caspase-3, Bax, and Bcl-2 in each group. **(D)** “*” *p* < 0.05, “**” *p* < 0.01 and “***” *p* < 0.001. Reproduced with permission from a previous publication (Luo et al., 2021).

Over recent years, there has been significant interest in exosomes as a means of intercellular information transmission; data indicates that exosomes might be able to regulate inflammation. In a previous study, Chen et al. (Xia et al., 2019) reported that mesenchymal stem cell (MSC)-derived exosomes regulated inflammation in degenerative NP cells *via* their inhibitory effect on NLRP3. Zhu et al. (2020) further demonstrated that MSC-derived exosomes reduced the IL-1β-induced secretion of multiple inflammatory factors as well as achieving the targeted inhibition of the MAPK signaling pathway by packaging *mircoRNA-142-3P*. Bone-MSC-derived exosomes were also reported to inhibit the expression of IL-1β and TNF-α and promote autophagy (Xiao et al., 2022). Luo et al. (2021) compared the effects of exosomes derived from normal and degenerative CEP stem cells on IDD and found that exosomes derived from normal CEP stem cells had a better effect in terms of promoting autophagy (Figure 3).

Although many natural anti-inflammatory products are produced by animals; most of these have very low levels of immunogenicity. However, considering their animal source, these natural anti-inflammatory products are subject to ethical issues; furthermore, the lack of stable donor sources that can maintain an industrial production scale represents a major obstacle to their clinical application.

### 3.2 Natural anti-inflammatory products derived from plants

Compared with animal-based agents, plant-derived anti-inflammatory substances are easier to obtain and there is a much wider range of products. Furthermore, a wide variety of natural substances have been derived from different plants and shown to have promising application prospects.

**FIGURE 4:**
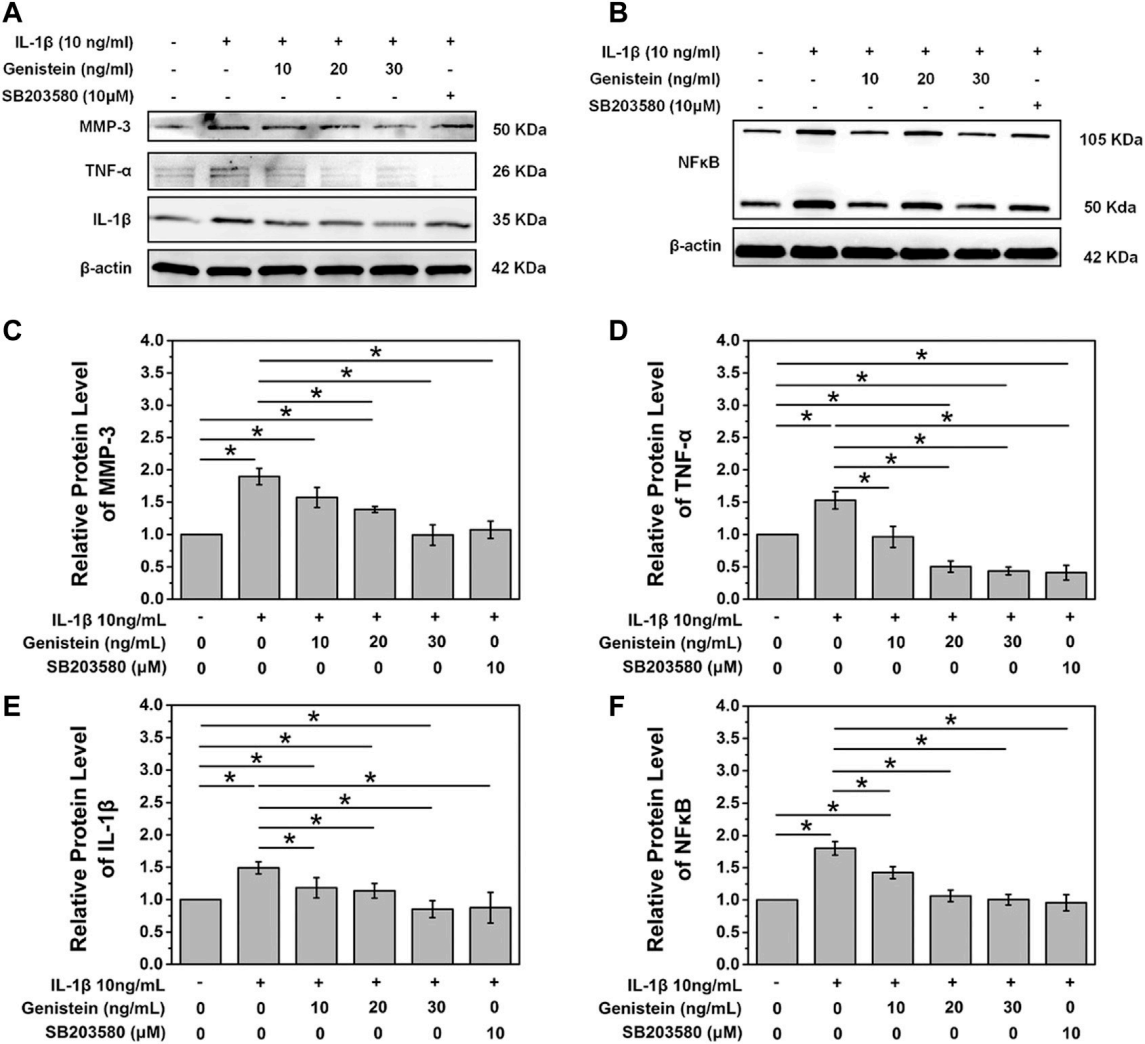
A comparison of the protein expression of MMP-3, TNF-α, and IL-1β in each group **(A)** A comparison of the protein expression of NF-κB in each group **(B)** Quantitative analysis of the protein levels of MMP-3 **(C)**, TNF-α **(D)**, IL-1β **(E)**, and NF-κB **(F)**, “*” *p* < 0.05. SB203580, a specific p38 MAPK inhibitor. Reproduced with permission from a previous publication (Ge et al., 2020).

**FIGURE 5:**
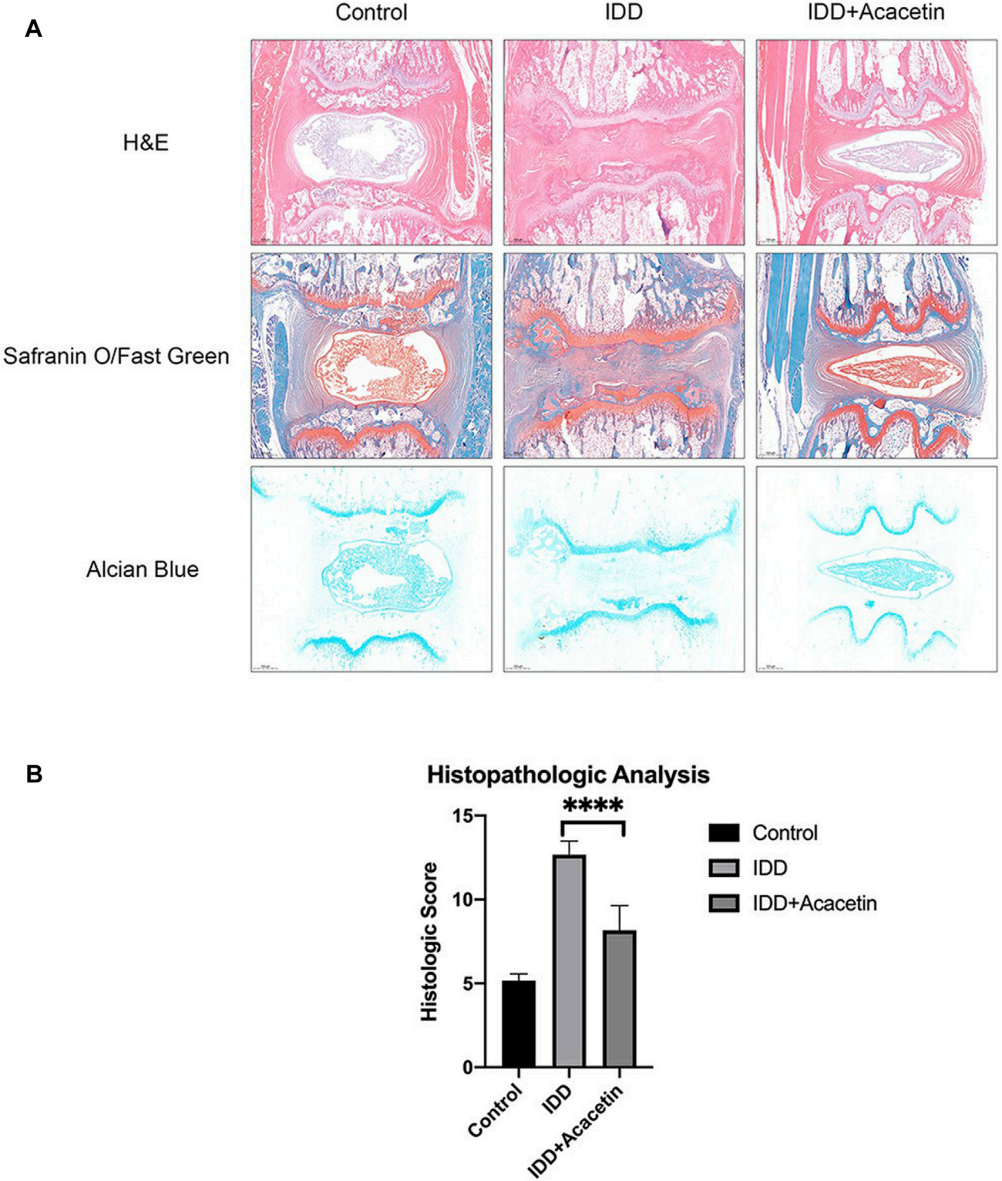
Images of IVD from rats in each treatment group stained with Safranin O/Fast Green and Alcian Blue in each group **(A)** A comparison of histological scores in each group **(B)**, “****” *p* < 0.0001. Reproduced with permission from a previous publication (Wang et al., 2020b).

Icariin is a type of flavonoid extracted from *Epimedium* and is believed to exert significant anti-inflammatory and antioxidant effects (Wang et al., 2022a; Wang et al., 2022b). Hua et al. (2018) reported that icariin inhibited activation of the NF-κB and MAPK pathways induced by IL-1β to suppress inflammation in degenerative NP. In another study, these authors found that icariin could effectively resist oxidative stress damage caused by hydrogen peroxide and maintain mitochondrial homeostasis (Hua et al., 2020). Shao et al. (2022) further found that icariin could prevent the degeneration and calcification of CEP by inhibiting cell apoptosis and ferroptosis. Baicalein, a flavonoid with an anti-inflammatory effect, is found in abundance in *Scutellaria baicalensis* (Tuli et al., 2020). In an *in vitro* study, baicalein effectively inhibited the expression of NO, IL-6, TNF-α and PEG2 in NP cells, but also reversed the overexpression of MMP-13 and ADAMTS-5 (Jin et al., 2019). Wogonin is another form of ketone extracted from *Scutellaria baicalensis*. Fang et al. (2018) reported the inhibitory effect of wogonin on IL-1β-induced inflammatory factors and enzymes that promote ECM degradation; in addition, these authors also observed that wogonin upregulated the expression of type II collagen. Genistein, a flavonoid extracted from soybean, has been shown to be effective in preventing osteoarthritis and osteoporosis (Wu and Liu, 2022). In IDD, genistein was shown to increase the secretion of type II collagen and aggrecan and reduce the expression of inflammatory factors by inhibiting the P38 MAPK pathway (Ge et al., 2020) (Figure 4). Naringin, as a citrus flavonoid, has received significant attention over recent years due to its immunomodulatory effect in the treatment of inflammation-related diseases (Zeng et al., 2018). Li et al. (Li K et al., 2016) reported that naringin inhibited the expression of TNF-α and MMP-13 and upregulated the expression of type II collagen and BMP-2 in degenerated human NP cells. In a subsequent study, these authors further showed that naringin alleviated TNF-α-induced inflammation and oxidative stress by enhancing autophagy (Chen et al., 2022). Quercetin is a natural flavonoid that is widely present in plants. Several previous studies have reported the application of quercetin in inflammation-related diseases (Dong et al., 2020; Yuan et al., 2020). Shao et al. (2021) reported that quercetin could inhibit initiation of the NF-κB signaling pathway by IL-1β and reduce the expression of a senescence associated secreted phenotype. As with quercetin, luteoloside is also a natural flavonoid that is widely present in plants. Lin et al. (2019) reported that luteolin inhibited the expression of multiple inflammatory factors in NP cells, protected IL-1β-induced ECM degradation, and inhibited apoptosis. In the mouse model of IDD, luteolin was shown to effectively alleviate the progression of degeneration. Other flavonoids from plants have been reported to modulate inflammation in IDD, including acacetin and apigenin. Wang et al. (2020b) reported the inhibitory effect of acacetin on inflammatory factors and its protective effect on the ECM *in vitro*; in an *in vivo* study, acacetin significantly reduced the progression of IDD (Figure 5). An *in vitro* study reported that apigenin may regulate inflammation by inhibiting TNF-α (Ding and Li, 2020). Xie et al. (2021) performed *in vivo* experiments and reported the activation of autophagy by apigenin and the alleviation of IDD.

**FIGURE 6:**
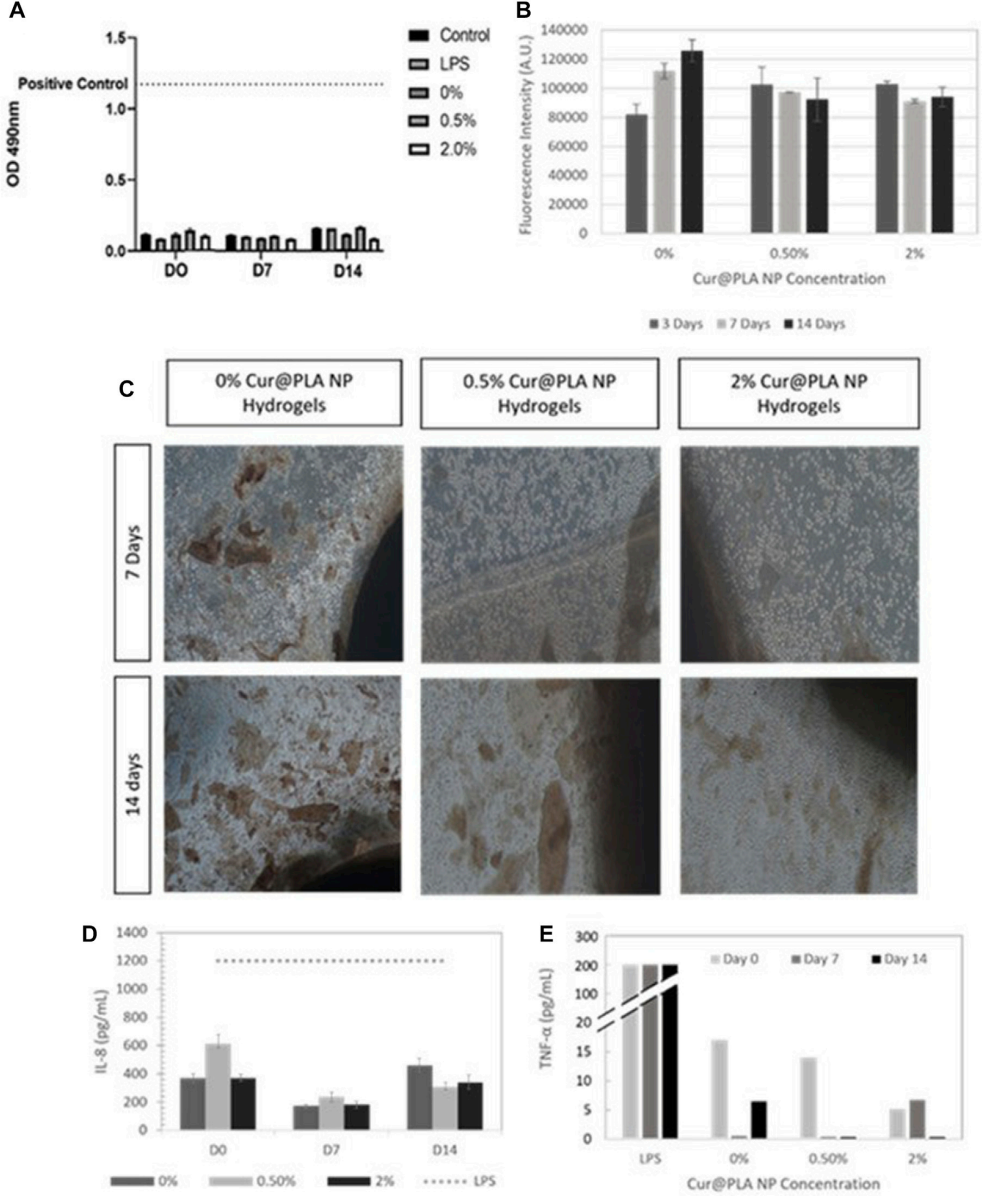
An evaluation of cytocompatibility of hydrogels containing different concentrations of curcumin by LDH **(A)** and Alamar blue **(B)**. Optical microscope images of hydrogel cultured cells containing different concentrations of curcumin at 7 and 14 days **(C)**. The levels of IL-8 **(D)** and TNF-α **(E)** produced by cells when exposed to hydrogels with different concentrations of curcumin. Reproduced with permission from a previous publication (Zamboni et al., 2022).

In addition to flavonoids, a significant number of natural anti-inflammatory substances also exist in the form of phenolics. Epigallocatechin-3-gallate (EGCG) is an active component of tea polyphenols. In recent years, EGCG has drawn significant attention due to its anti-inflammatory, anti-oxidation and anti-cancer effects (Li R et al., 2022; Mokra et al., 2022). Krupkova et al. (2014) reported that EGCG could reduce the inflammatory response triggered by IL-1β *in vitro* and observed a reduced level of radicular pain in a rat model *in vivo.* In their subsequent study, these authors further reported that EGCG could resist oxidative stress in IDD by maintaining mitochondrial stability (Krupkova et al., 2016). Tian et al. (2020) demonstrated that EGCG inhibited cellular inflammation and apoptosis in human degenerative NP cells by inhibiting the activation of NLRP3. Resveratrol is a natural phenolic antitoxin that is widely found in grapes, peanuts, and other plants (Meng et al., 2021; Ren et al., 2021). Resveratrol has been widely used in the fields of food processing, healthcare and medicine. Li et al. (2008) reported that resveratrol could slow down IDD by inhibiting the downstream signaling factors of IL-1. Jiang et al. (2019) further reported that resveratrol could inhibit the apoptosis of NP cells triggered by IL-1β. Similarly, Wu et al. (2021) reported that resveratrol reduced IL-6 expression and inhibited the phosphorylation of Janus kinase 1 downstream, and signal transducer and activator of transcription 3. Honokiol is a phenolic substance extracted from *Magnolia*. In another study, Wang et al. (2018a) reported the activation of honokiol by Sirtuin-3, a protein that maintains mitochondrial stability. Tang et al. (2018) further demonstrated that honokiol could alleviate inflammation, oxidative stress and apoptosis in IDD by inhibiting the TXNIP/NLRP3/caspase-1/IL-1β signaling pathway and the activation of NF-kB and JNK. Moreover, honokiol was also able to upregulate the expression of type II collagen. Curcumin is a natural pigment that has been widely used in food processing; there is strong evidence for the medical value of this product (Zia et al., 2021). Kang et al. (2019) reported that curcumin activated autophagy and alleviated the progression of IDD in an *in vivo* rat model. Zamboni et al. (2022) further designed an alginate/gelatin hydrogel coated with curcumin nanoparticles and achieved significant TNF-α inhibition (Figure 6). Coumarin is also a spice and has promising medical applications (Al-Warhi et al., 2020). Su et al. (2019) reported the significant inhibitory effects of isoazinine, as a coumarin compound, on a variety of inflammatory factors and MMPs in human degenerative NP cells. Both sesamin and mangiferin have been extracted from common food raw materials, and their medicinal value has been gradually explored. Li et al. (Li N et al., 2016) reported that sesamin inhibited LPS-induced ECM-catabolic enzymes and inflammatory factors in a dose-dependent manner *in vitro*. In a subsequent *vivo* study, the injection of sesamin into the degenerative discs of a rat model achieved a potent protective effect against IDD (Li and Lv, 2020). As with mangiferin, Yu et al. (2021) reported that mangiferin has anti-inflammatory and anti-oxidative properties and that the maintenance of mitochondrial stability can influence IDD *in vitro*; in a subsequent animal model, the local injection of mangiferin effectively alleviated the progression of degeneration.

Berberine is an alkaloid derived from *Coptidis rhizoma*, a traditional form of Chinese medicine, which has antibacterial and anti-inflammatory effects (Song et al., 2020). Many studies have reported that berberine can alleviate cell apoptosis, ECM degradation, and oxidative stress damage, caused by inflammatory factors (Chen et al., 2018; Lu et al., 2019; Huang et al., 2022). Similarly, Wei et al. (2020) reported that oxymatrine, a matrine extract, could relieve inflammation in IDD by inhibiting the TLR4/NF-κB pathway. In another study, these authors reported the significant inhibition of IL-1β-induced IDD progression by oxymatrine liposomes in a mouse model (Wang et al., 2020a). Celastrol and glycyrrhizic acid are terpenoids extracted from traditional Chinese medicine. Chen et al. (2017) reported the inhibitory effect of celastrol on a variety of downstream inflammatory factors induced by IL-1β. Similarly, Liu et al. (2019) reported that glycyrrhizic acid could attenuate IL-1β-induced inflammation by inhibiting the high-mobility group box-1 gene. Fucoidan, a polysaccharide extracted from algae, has also been shown to inhibit inflammation in IDD (Yu et al., 2022).

Although the anti-inflammatory effects of plants were recognized long before the rapid development of modern medicine, the extraction of their active ingredients, and the exploration of their mechanisms, still require significant experimentation. In addition, a set of standard and objective evaluation criteria for the anti-inflammatory effect of IDD is still required.

## 4 Conclusion and outlook

IDD is a common degenerative disease that is a major contributor to LBP and disc herniation. IDD has a negative impact on a patient’s quality of life and results in significant social and financial losses. Natural products can be used as lead structures as well as a starting point when creating manufactured products to cure disc degeneration. However, natural products are more complex and variable than artificial products; thus, a drug development model aiming to identify a single active ingredient of artefacts may not be suitable for in-depth studies of natural products (Caesar and Cech, 2019). Even though natural products are highly promising for the treatment of IDD, it is still difficult to use these produces clinically. First, the precise mechanisms underlying the onset and progression of IDD have yet to be fully elucidated; these mechanisms are connected, thus creating a vicious cycle. Second, it is challenging and not yet ideal to recreate the pathogenic process of human IDD in an *in vivo* experimental model. Finally, even though natural products have demonstrated a remarkable and promising potential for use in the treatment of IDD, several repeatable trials and exploratory investigations are required to fully understand their cytotoxicity and genotoxicity. In addition, because the IDD features three biological aspects: the AF, NP, and EP; thus, treating IDD successfully should not be restricted to just one of these structures.

The following areas should be investigated further: 1) a deeper investigation of the pathogenic mechanisms underlying the emergence and progression of IDD is necessary, thus offering a clearer roadmap for the creation of natural remedies as well as a theoretical foundation for the replication of IDD in animal models; 2) a thorough understanding of the local metabolic status of NP, AF, and CEP cells during the use of natural products to treat IDD, as this may provide therapeutic recommendations for various stages of the degenerative process; 3) there are still many therapeutic options that have not been explored, and researchers need to keep exploring and generating new treatments for IDD, and 4) it is important that we investigate more ideal medicine dosages and effective delivery systems and improve the industrial production of natural products to guarantee consistency in quality across batches.
